# Decoding the tumor-aging axis: from bench to clinical

**DOI:** 10.3389/fimmu.2026.1823423

**Published:** 2026-07-10

**Authors:** Yuxin Wang, Guitong Lv, Xinyue Gao, Zheng Jin, Jiani Huang, Zhongyu Wang

**Affiliations:** 1 School of Pharmacy and Bioengineering, Chongqing University of Technology, Chongqing, China; 2 Institute of Cancer, Xinqiao Hospital, Third Military Medical University, Chongqing, China; 3 Institute of Immunological Innovation and Translation, Chongqing Medical University, Chongqing, China

**Keywords:** aging and cancer, cellular senescence, immunosenescence, senescence-associated secretory phenotype, tumor-aging axis

## Abstract

Population aging is a major global health challenge and a principal risk factor for cancer. Aging does not simply increase mutational burden; it reshapes tissue homeostasis across genetic, epigenetic, metabolic, immune, and systemic dimensions. Genomic instability, epigenetic drift, mitochondrial dysfunction, and metabolic rewiring collectively establish a tumor-permissive landscape that enhances clonal diversification and lowers the threshold for malignant transformation. Concurrently, accumulation of senescent cells and the senescence-associated secretory phenotype (SASP) remodel the microenvironment, promote immune suppression, and weaken tumor surveillance, further exacerbated by immunosenescence and gut microbiota dysbiosis. Importantly, cancer progression feeds back to accelerate organismal aging. Tumor burden and therapy-induced stress destabilize hematopoietic and non-hematopoietic stem cell niches, disrupt systemic metabolic homeostasis, and induce neuroendocrine reprogramming, thereby amplifying multi-organ functional decline. Aging and cancer therefore constitute a bidirectional and self-reinforcing network rather than a linear cause-effect relationship. In this review, we synthesize mechanistic, clinical, and translational evidence defining the tumor-aging axis and discuss emerging strategies aimed at interrupting this pathogenic cycle.

## 1 Introduction

Malignant tumors remain a leading cause of mortality worldwide and constitute a major component of the global disease burden (1). In parallel, the global population is undergoing rapid demographic aging, with a sustained expansion of individuals aged 65 years and older. This demographic shift, together with the well-established association between aging and cancer risk, has driven a substantial increase in global cancer incidence. Currently, approximately 60 percent of newly diagnosed cancers occur in individuals aged 65 years or older (2, 3). This age-dependent rise in cancer incidence is observed across multiple malignancies, including breast, colorectal, and prostate cancers. Aging is therefore recognized not only as a major risk factor for tumorigenesis but also as an important determinant of adverse clinical outcomes in malignant disease (4–7).

At the molecular and cellular levels, tumorigenesis and organismal aging share a set of fundamental biological hallmarks, including genomic instability, epigenetic dysregulation, chronic inflammation, stem cell exhaustion, and alterations of the microbiome (8, 9). These processes are not independent. Instead, they are interconnected and form a self-reinforcing network in which aging promotes tumor initiation and progression, while cancer development and its treatment reciprocally accelerate systemic aging. This bidirectional interplay provides the conceptual basis for the tumor-aging axis.

Aging contributes to tumor development through convergent and multilayered mechanisms. Although senescence initially acts as a tumor-suppressive barrier in premalignant tissues, the accumulation of senescent cells and age-associated DNA damage may also increase genomic instability and clonal diversity (10). Aging also reshapes the epigenetic and metabolic architecture of tissues. Epigenetic drift and chromatin remodeling progressively reduce regulatory fidelity and enhance transcriptional plasticity, thereby lowering the threshold for oncogenic reprogramming (11, 12). Concurrently, mitochondrial dysfunction and age-associated metabolic rewiring establish a pro-inflammatory and bioenergetically altered milieu that favors malignant transformation through mitonuclear stress signaling and redox imbalance (13). Persistent secretion of inflammatory mediators through the senescence-associated secretory phenotype (SASP) further remodels the tumor microenvironment and reinforces oncogenic signaling (14). In parallel, immunosenescence and microbiota dysbiosis weaken systemic immune surveillance and tissue homeostasis (15, 16). Together, these interconnected processes create a tumor-permissive ecological landscape that facilitates malignant initiation and progression.

Conversely, tumors and anticancer therapies reciprocally accelerate systemic aging. Tumor-derived inflammatory mediators and circulating factors induce senescence in distant tissues and destabilize stem cell niche integrity beyond the hematopoietic system, thereby compromising regenerative capacity (17, 18). Malignant progression is further accompanied by metabolic competition and neuroendocrine reprogramming, which amplify multi-organ functional decline through sustained stress signaling and endocrine imbalance (19). In parallel, conventional anticancer treatments, although effective in controlling malignant cells, frequently induce DNA damage and therapy-associated senescence in normal tissues, contributing to premature aging phenotypes (20). These bidirectional interactions establish a self-reinforcing cycle in which cancer and aging converge at molecular, cellular, and systemic levels.

Understanding the regulatory principles governing the tumor-aging axis is therefore essential for improving cancer prevention and treatment, particularly in the context of an aging population. A persistent challenge in this field is the underrepresentation of aged populations in both preclinical models and clinical studies, which limits the generalizability of current evidence on the tumor-aging axis. This review synthesizes current evidence delineating the mechanisms by which aging drives tumor initiation and progression, examines how tumors and their therapies accelerate systemic aging, and discusses emerging translational strategies aimed at disrupting this bidirectional circuit. Given the breadth of aging-associated hallmarks, the discussion focuses on those most directly implicated in the tumor-aging axis and emphasizes their functional convergence rather than treating them as isolated processes. Deciphering the tumor-aging axis may ultimately open new avenues for cancer prevention, identify novel therapeutic targets, and improve both clinical outcomes and quality of life for older patients with cancer.

## 2 Mechanisms of aging-driven tumor development

Organismal aging drives oncogenesis through a complex interplay of molecular and systemic dysregulations. At the cellular level, genomic instability and epigenetic drift expand transcriptional plasticity, effectively lowering the biological threshold for malignant reprogramming. This intrinsic vulnerability is further exacerbated by mitochondrial dysfunction and metabolic rewiring, which fuel oxidative stress and establish an energetic landscape conducive to tumor survival. Beyond these cell-intrinsic changes, the accumulation of senescent cells and their associated secretory phenotype (SASP) progressively remodel the local tissue architecture into a pro-inflammatory niche. When coupled with systemic immunosenescence and gut microbiota dysbiosis, these multi-scale processes collectively undermine host surveillance, transforming homeostatic tissues into a permissive environment for tumor initiation, clonal evolution, and immune evasion.

### 2.1 Genomic instability in aging fuels tumorigenesis

Age-related genomic instability represents a fundamental driver of tumorigenesis. With advancing age, progressive telomere shortening triggers DNA damage responses (DDR) and activates p53 signaling, which normally induces cellular senescence to eliminate potentially malignant cells (21, 22). In premalignant cells, senescence serves as an important tumor-suppressive mechanism, in which coordinated activation of the p53/p21 and p16INK4A/RB pathways enforces stable cell-cycle arrest and limits the expansion of genomically unstable cells (23, 24). In the early stages of tumor development, this senescence-associated barrier restrains malignant conversion by maintaining durable growth arrest. However, persistent checkpoint activation may also impose selective pressure, favoring the emergence of clones capable of bypassing senescence-associated control pathways. Once this barrier is overcome, telomere dysfunction no longer acts mainly as a tumor-suppressive trigger, but instead becomes a source of ongoing genomic instability, promoting chromosome mis-segregation, mutation accumulation, and clonal evolution, thereby accelerating tumor progression (25, 26).

In human hepatocellular carcinoma, tumor cells maintain critically short telomeres through mechanisms such as telomerase reactivation. This telomere state is insufficient to trigger senescence but is sufficient to sustain chronic genomic instability that promotes malignant progression (27, 28). Consistent with this observation, population-based studies demonstrate that shorter telomere length is associated with increased risk of multiple cancers, including lung, bladder, and gastrointestinal malignancies (29, 30). Persistent DNA damage arising from telomere dysfunction predisposes cells to inactivating mutations in key tumor suppressor genes such as TP53, thereby driving uncontrolled proliferation. Such alterations not only remove critical restraints on cell-cycle progression but also weaken senescence-associated checkpoint control, enabling damaged cells to expand despite ongoing genomic stress (31, 32). In colorectal cancer, such alterations are linked to sustained activation of the AKT pathway, upregulation of programmed death ligand 1 (PD-L1), and enhanced immune escape (33, 34).

Beyond telomere attrition, aging further amplifies genomic instability through progressive impairment of DNA repair pathways. Senescent cells frequently exhibit homologous recombination deficiency (HRD), characterized by epigenetic dysregulation and loss of DNA repair protein function, particularly involving BRCA1 (35, 36). Pan-cancer analyses consistently reveal higher HRD scores in older patients, establishing aging as a key risk factor for HRD-associated phenotypes, including increased tumor mutational burden and microsatellite instability (37, 38). Notably, HRD-associated tumors often develop an immunosuppressive microenvironment, contributing to resistance to immune checkpoint inhibitors (ICIs). This phenomenon is exemplified by the limited survival benefit of ICIs observed in BRCA1-mutant triple-negative breast cancer (39). Importantly, combination strategies integrating poly (ADP-ribose) polymerase (PARP) inhibitors with ICIs have shown improved efficacy, highlighting a rational therapeutic approach to overcome age-associated genomic instability and immune resistance (40).

Together, telomere dysfunction and HRD-related DNA repair impairment cooperate to promote genomic instability in aging tissues. In this context, the role of senescence in tumorigenesis is not unidirectional: senescence initially acts as a barrier to malignant transformation, whereas persistence of senescence-associated stress or escape from checkpoint control may facilitate genomic instability, clonal evolution, and subsequent tumor progression.

### 2.2 Epigenetic drift and transcriptional reprogramming

**Figure 1:**
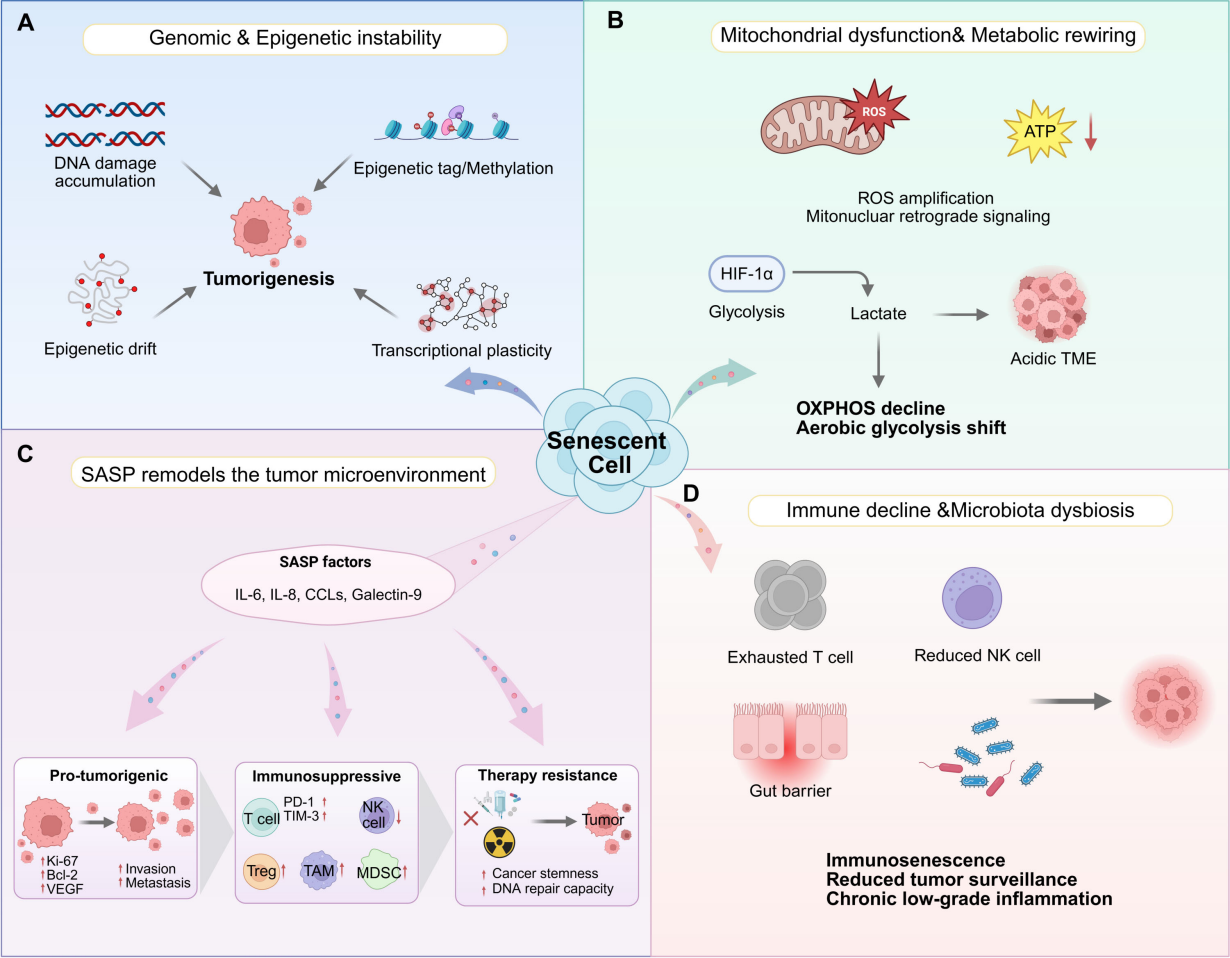
Mechanisms of aging-driven tumorigenesis. **(A)** Genomic and epigenetic instability. Accumulating DNA damage and epigenetic alterations, including chromatin remodeling and transcriptional dysregulation, promote transcriptional plasticity and clonal diversification, creating a permissive environment for tumor initiation. **(B)** Mitochondrial dysfunction and metabolic rewiring. Aging-related mitochondrial dysfunction and increased oxidative stress drive a shift toward aerobic glycolysis (the Warburg effect), establishing a pro-inflammatory and nutrient-deprived tumor microenvironment. **(C)** SASP remodels the tumor microenvironment. The accumulation of senescent cells releases pro-inflammatory factors through the SASP, contributing to chronic inflammation and remodeling of the tumor microenvironment, which supports tumorigenesis. **(D)** Immune and microbial dysbiosis. Age-associated immune decline (immunosenescence) and gut microbiota imbalance impair immune surveillance, further facilitating tumor initiation and progression. These interconnected processes lower the threshold for oncogenic transformation, transforming otherwise homeostatic tissues into environments permissive to malignant initiation.

Unlike genetic mutations, epigenetic alterations accumulate progressively prior to overt malignant transformation and modulate cell fate in a dynamic and potentially reversible manner. During aging, DNA methylation patterns undergo a characteristic epigenetic drift, marked by global hypomethylation alongside focal hypermethylation at specific CpG islands (41, 42). Global hypomethylation predominantly affects repetitive elements and transposon-rich regions, leading to aberrant activation of these sequences, genomic instability, and increased tumor susceptibility. In contrast, hypermethylation of promoter CpG islands in gene-rich regions can silence key regulatory genes involved in cell cycle control, DNA repair, and tumor suppressor pathways (43). This bidirectional imbalance reflects a progressive decline in epigenetic precision and has been conceptualized as an age-associated increase in epigenetic entropy (12). Rather than representing passive molecular noise alone, this age-associated methylomic drift functionally destabilizes lineage-restricted transcriptional programs and lowers the threshold for selection of premalignant clones (44, 45) (**Figure 1**).

Beyond DNA methylation, alterations in chromatin organization and histone modifications further destabilize transcriptional regulation. Senescent cells exhibit reduced core histone content, relaxation of heterochromatin, and loss of repressive marks such as H3K9me3 and H3K27me3 (46, 47). These changes permit aberrant activation of previously silenced genomic regions and disrupt higher-order chromatin architecture, thereby affecting three-dimensional genome organization and global transcriptional control (48). Dynamic shifts in histone acetylation also alter expression of genes governing cell cycle progression, DNA repair, and apoptosis (49). Together, these chromatin alterations disrupt higher-order chromatin architecture and transcriptional control, increase transcriptional variability, weaken maintenance of cellular identity, and enhance phenotypic plasticity, thereby facilitating oncogenic reprogramming.

An additional and increasingly recognized layer of age-associated reprogramming is epitranscriptomic regulation, particularly N^6^-methyladenosine (m^6^A), the most prevalent internal modification in eukaryotic mRNA. m^6^A is dynamically installed by writer complexes such as METTL3/METTL14/WTAP, removed by the erasers FTO and ALKBH5, and interpreted by reader proteins including YTH domain-containing proteins and IGF2BP family members to regulate RNA stability, splicing, translation, and decay (50, 51). During aging, altered m^6^A homeostasis has been increasingly linked to senescence-associated programs and disruption of cellular homeostasis, including pathways involving p53, NF-kB, and inflammatory remodeling (52, 53). In cancer, dysregulated m^6^A signaling can stabilize oncogenic transcripts, support metabolic and hypoxic adaptation, and reshape the tumor microenvironment by influencing immune-cell function and immune checkpoint signaling (54, 55). The consequences of m^6^A remodeling are context-dependent, and individual regulators may exert tumor-promoting or tumor-suppressive effects depending on tissue and disease stage (56). Thus, epitranscriptomic remodeling should be considered an integral component of the tumor-aging axis, linking age-associated epigenetic drift to post-transcriptional control of cellular plasticity, immune evasion, and therapeutic response.

Dysregulation of non-coding RNAs represents an additional layer of epigenetic remodeling. lncRNAs, miRNAs, and circular RNAs regulate gene expression through recruitment of chromatin-modifying complexes or post-transcriptional modulation of target mRNAs (57). For example, miR-455-3p has been linked to lifespan and cognitive function in animal models, and its downregulation promotes proliferation and invasion in cancer cells (58). While individual non-coding RNAs exert context-specific effects, aging is characterized by broad remodeling of regulatory RNA networks, which amplifies transcriptional fluctuations and reinforces oncogenic signaling circuits (59). These non-coding RNA networks do not function in isolation but intersect with DNA methylation, chromatin remodeling, and m^6^A-dependent RNA regulation to reinforce aberrant cell-state transitions during tumor evolution.

In summary, aging-associated regulatory drift extends beyond DNA methylation and chromatin remodeling to include epitranscriptomic reprogramming and broad rewiring of non-coding RNA networks. These reversible yet cumulative alterations increase transcriptional noise, erode cellular identity, and create a permissive substrate for clonal selection, malignant progression, immune escape, and therapeutic adaptation.

### 2.3 Mitochondrial dysfunction and metabolic rewiring

Epigenetic instability and mitochondrial dysfunction are functionally interconnected during aging and may form a reinforcing cycle. Telomere damage is frequently accompanied by mitochondrial impairment, reduced electron transport chain efficiency, and excessive reactive oxygen species (ROS) production (60). Elevated ROS levels induce oxidative damage to DNA, proteins, and lipids, promote mitochondrial DNA mutations, and sustain chronic oxidative stress. ROS accumulation can also activate oncogenic pathways such as HIF-1α, enhancing glycolysis and angiogenesis and thereby supporting tumor growth and dissemination (61, 62). Oxidative DNA lesions further contribute to mutagenesis, potentially impairing key regulators such as p53 or activating pathways such as RAS, thereby promoting malignant transformation (63). Thus, mitochondrial dysfunction functions not only as a metabolic defect but also as a central node linking oxidative stress, genomic instability, and oncogenic signaling.

Aging is accompanied by a progressive loss of metabolic flexibility, characterized by diminished oxidative phosphorylation and a relative increase in aerobic glycolysis (64, 65). This shift is associated with sustained activation of signaling pathways including PI3K-AKT-mTOR (66). Increased ROS levels in aged tissues can stabilize HIF-1α and further bias cellular metabolism toward glycolysis (67). These alterations do not directly induce cancer; rather, they lower the threshold for a Warburg-like metabolic transition, predisposing cells to oncogenic metabolic reprogramming.

Mitochondrial dysfunction also reshapes lipid and amino acid metabolism. Senescent cells upregulate cPLA2α via MAPK and STAT signaling, promoting lipid droplet formation and enhanced lipid synthesis. Tumor cells increase lipid uptake and storage to support membrane biogenesis and energy demands (68, 69). Age-associated disturbances in glutamine metabolism can impair T-cell function and weaken anti-tumor immunity (70). Importantly, mitochondrial dysfunction also engages mitochondrial retrograde signaling. Reduced respiratory chain activity or mtDNA damage triggers changes in ROS levels, Ca2^+^lux, and NAD^+^/ATP ratios, activating nuclear pathways including HIF-1α, NF-κB, ATF4, and cGAS-STING (71, 72). These signals reprogram nuclear gene expression, promote inflammatory mediator production, alter metabolic enzyme expression, influence epigenetic states, and thereby reinforce age-related transcriptional instability and tumor-associated signaling networks.

Collectively, aging-associated mitochondrial dysfunction establishes a tissue context characterized by chronic inflammation and metabolic imbalance. Although insufficient to initiate malignancy independently, this state lowers the threshold for metabolic transformation, amplifies oncogenic signaling, and impairs immune surveillance. In this regard, mitochondrial dysfunction serves as a metabolic pre-adaptation platform that cooperates with genomic and epigenetic instability to accelerate malignant evolution.

### 2.4 SASP-mediated tumor progression

The SASP comprises a heterogeneous array of cytokines, chemokines, growth factors, and proteases that play a central role in establishing a tumor-promoting microenvironment. By enhancing tumor cell proliferation, invasion, metastasis, and therapy resistance, the SASP actively drives cancer progression (73).

Key SASP components directly stimulate malignant behavior. IL-6, for example, activates the JAK-STAT3 pathway, leading to upregulation of anti-apoptotic proteins such as Bcl-2 and Bcl-xL and induction of epithelial-mesenchymal transition, thereby enhancing tumor proliferation and invasiveness. Inhibition of this pathway suppresses tumor growth and promotes apoptosis, as demonstrated in glioma models (74, 75). Beyond IL-6, additional SASP factors, including growth differentiation factor 15 (GDF15) and cyclin B2 (CCNB2), contribute to tumor development, migration, and self-amplification of the SASP in colorectal cancer and glioma (76, 77), underscoring the intrinsic tumor-promoting capacity of the senescent secretome.

A major pro-tumorigenic function of the SASP lies in its ability to induce immunosuppression. In pancreatic ductal adenocarcinoma, senescent cancer-associated fibroblasts secrete SASP factors that suppress CD8^+^ T cell function and diminish immunotherapy efficacy (78). Similarly, in melanoma, senescent fibroblasts release IL-6, MMP3, and CCL family chemokines that activate the NF-κB signaling, thereby promoting immune evasion and tumor growth (79, 80). The SASP also contributes directly to resistance against immunotherapy. In lung adenocarcinoma, tumor-derived exosomal tsRNA induces fibroblast senescence and subsequent secretion of galectin 9, leading to resistance to anti-PD-L1 therapy (81).

In addition to immune modulation, the SASP plays a key role in therapy resistance. Therapy-induced senescent cells maintain active metabolism under cytotoxic stress and continuously release SASP factors that enhance the invasion and metastatic potential of residual tumor cells, ultimately contributing to treatment failure (82, 83). In breast cancer, myofibroblast-like cancer-associated fibroblasts confer chemoresistance by secreting IL-6 and IL-8, which enhance cancer stemness and attenuate drug cytotoxicity (84).

Collectively, these findings position the SASP as a central orchestrator of tumor progression, immune suppression, and therapeutic resistance.

### 2.5 Immunosenescence undermines immune surveillance

Immunosenescence, defined as the age-associated decline in immune competence accompanied by chronic low-grade inflammation, represents a critical mechanistic link between aging and tumor progression. Rather than reflecting a simple reduction in immune activity, aging reshapes tumor immune surveillance at multiple levels, including immune recognition, antigen presentation, effector-cell fitness, and local tissue immune defense. In the framework of cancer immunoediting, these changes weaken the elimination phase, destabilize tumor-immune equilibrium, and ultimately facilitate immune escape, thereby promoting tumor progression and therapeutic resistance (85–87).

Decline of CD8^+^ T-cell function lies at the core of impaired antitumor immunity in aged hosts. Thymic involution reduces the output of naive T cells, limiting the diversity of the T cell receptor repertoire available for tumor recognition (88). Aging further promotes T cell exhaustion through upregulation of inhibitory receptors such as PD-1 and Tim-3, partly mediated by IL-6/STAT3 signaling (89). Clinically, accumulation of senescent (CD28^-^ CD57^+^) CD8^+^ T cells correlate with poor prognosis in elderly patients with colorectal cancer (90). Recent work in aged mice further shows that NK-cell transfer can overcome resistance to PD-(L)1 therapy, highlighting a functional link between age-related NK-cell impairment and reduced immunotherapeutic responsiveness (91) Recent studies have identified a dysfunctional CD8^+^ T cell subset characterized by high expression of PD-1 and TOX and low expression of TCF7, which is enriched in aged tumor microenvironments and exhibits severely impaired cytotoxicity. These findings suggest that aging not only reduces effector-cell abundance but also actively reprograms CD8^+^ T cells toward dysfunctional states that favor immune escape. Targeting upstream epigenetic regulators of this population may therefore offer a strategy to restore antitumor immunity (92). Additionally, aging impairs local immune surveillance by reducing the abundance and function of tissue-resident memory T cells (TRM). In aged mice, this defect has been attributed to age-associated upregulation of the E3 ubiquitin ligase BFAR in CD8^+^ T cells, which suppresses JAK-STAT signaling and compromises tissue-specific immune defense. Loss of TRM populations further weakens localized antitumor immunity and contributes to immune escape within aging tissues (93). In addition to adaptive T-cell dysfunction, aging may also compromise innate immune surveillance mediated by natural killer (NK) cells, thereby weakening the early recognition and elimination of transformed cells (94, 95). Recent work in aged mice further shows that NK-cell transfer can overcome resistance to PD-(L)1 therapy, highlighting a functional link between age-related NK-cell impairment and reduced immunotherapeutic responsiveness (96).

Aging also profoundly reshapes hematopoiesis, promoting systemic immunosuppression that supports tumor growth. Age-related clonal hematopoiesis, driven by mutations in genes such as DNMT3A, TET2, and ASXL1, leads to expansion of myeloid-biased progenitors and establishes a pro-inflammatory and immunosuppressive baseline (97, 98). In parallel, aging induces a myeloid bias characterized by increased production of granulocyte macrophage progenitors. These progenitors readily differentiate into immunosuppressive myeloid populations within the tumor microenvironment (99, 100). Persistent IL-1 signaling in aged hosts drives recruitment of myeloid progenitor-like cells, which subsequently differentiate into M2-like tumor-associated macrophages that secrete IL-10 and TGF-β to suppress CD8^+^ T cell function (101, 102). Blockade of IL-1 signaling reverses this cascade and delays tumor progression in aged lung cancer models (103, 104). Thus, aging does not merely blunt immune effector function; it also actively remodels the hematopoietic and myeloid compartments into a tumor-permissive state.

In addition to myeloid expansion, aging increases populations of myeloid-derived suppressor cells, which inhibit T cell function through mechanisms including PD-L1 engagement and arginase 1-mediated nutrient depletion (105, 106). Dendritic cell (DC) function is also compromised, with reduced major histocompatibility complex class II expression, contraction of cDC1 subsets, and impaired migration, collectively limiting effective CD8^+^ T cell priming (107, 108). Importantly, these defects remain plastic, as DC activators have been shown to restore antitumor immunity (109, 110). Beyond functional decline, aging can actively reprogram CD8^+^ T cells into suppressive phenotypes. Accumulation of CD39^+^CD73^+^CD8^+^ T cells promotes adenosine-dependent inhibition of CD4^+^ T cell responses, further reinforcing immune suppression and tumor progression (111). Taken together, aging shifts antitumor immunity from a surveillance-dominant state toward an exhausted and suppressive immune ecosystem, in which impaired cytotoxic function, defective antigen presentation, and myeloid remodeling cooperate to facilitate immune escape and tumor progression.

Importantly, age-related immunosuppression is not irreversible. A phase I trial in head and neck squamous cell carcinoma (COIS-01) demonstrated that a combination of senolytic drugs and anti-PD-1 therapy can effectively clear senescent immune cells (particularly senescent CD4^+^ T cells), enhance intratumoral T cell infiltration, and achieve a pathological response rate of 33.3% (112). These findings provide clinical proof of concept that therapeutic targeting of immunosenescence may restore tumor immune surveillance and improve antitumor efficacy in aged patients.

### 2.6 Gut microbiota dysbiosis promotes tumor progression

Age-associated dysbiosis of the gut microbiota represents an additional mechanism by which aging promotes tumor progression. With advancing age, beneficial microbial taxa such as Bifidobacterium decline, whereas opportunistic pathogens, including members of the Enterobacteriaceae, expand (113). This ecological imbalance promotes tumorigenesis and metastasis through integrated metabolic, barrier, and immune pathways.

Dysbiosis leads to the accumulation of carcinogenic metabolites such as deoxycholic acid (DCA), which directly stimulate tumor cell proliferation and suppress apoptosis (114). Supporting a causal role, fecal microbiota transplantation from aged donors enhances colon tumorigenesis in murine models (115). Specific pathogens, including Escherichia coli C17, disrupt epithelial tight junctions, impair intestinal barrier integrity, and facilitate translocation of bacteria and tumor cells to the liver, potentially contributing to the high incidence of liver metastasis in older patients with colorectal cancer (116). In parallel, microbial metabolites drive differentiation of Th17 cells and production of IL-17, sustaining chronic inflammation that remodels the tumor microenvironment and supports cancer progression (117, 118).

Importantly, the contribution of age-associated dysbiosis may also be modifiable. In preclinical models, microbiota-directed interventions such as fecal microbiota transplantation have been shown to suppress colorectal tumor progression by reversing microbial imbalance, attenuating intestinal inflammation, and enhancing antitumor immune responses (119). Emerging evidence further suggests that age-related differences in gut microbiota composition can influence therapeutic responsiveness. In particular, specific aging-enriched microbial configurations have been associated with favorable responses to immune checkpoint blockade, whereas age-associated gut microbiome changes have also been shown to affect the efficacy of tumor immunomodulatory treatments in aged mice (120, 121). Together, these findings indicate that gut dysbiosis in aging is not only a contributing mechanism of tumor progression, but also a potentially actionable component of the tumor-aging axis.

Collectively, age-associated gut microbiota dysbiosis promotes tumor progression through intertwined metabolic, barrier, and immune mechanisms, thereby linking microbial imbalance to the broader process of aging-driven tumorigenesis.

### 2.7 An integrative framework linking aging hallmarks to tumor biology

Rather than acting as isolated defects, the major hallmarks of aging converge on a limited set of tumor-relevant biological outputs. Genomic instability and epigenetic dysregulation expand clonal diversity and transcriptional plasticity, creating a permissive substrate for malignant evolution. Mitochondrial dysfunction, metabolic rewiring, and persistent inflammatory signaling further support survival under stress and remodel the tissue microenvironment. In parallel, senescent cell accumulation, immunosenescence, and microbiota dysbiosis weaken immune surveillance and reinforce stromal and immune conditions that favor tumor persistence. Viewed together, these processes function across molecular, cellular, microenvironmental, and systemic levels to promote four interrelated consequences of tumor progression: clonal evolution, phenotypic plasticity, immune escape, and therapeutic adaptation.

## 3 Mechanisms of tumor-driven acceleration of aging

Tumor development exerts profound systemic effects that extend beyond local tissue invasion. Through chronic inflammation, metabolic disruption, and therapy-induced damage, tumors actively accelerate systemic aging. These processes reshape hematopoiesis, induce senescence in distant organs, and culminate in multisystem functional decline. Importantly, tumor-induced neuroendocrine reprogramming and metabolic competition exacerbate aging-related dysfunction in multiple organs, further impairing systemic homeostasis. These interactions between tumor progression and aging underscore the bidirectional nature of the tumor-aging axis, where tumor growth not only accelerates aging but also contributes to the frailty and compromised function observed in older cancer patients.

### 3.1 Tumor-induced hematopoietic aging

Tumors profoundly remodel the hematopoietic niche through targeted disruption of bone marrow homeostasis, thereby accelerating hematopoietic aging (122). Myeloid malignancies drive niche inflammation via tumor-derived IL-1β. IL-1β polarizes LepR^+^ mesenchymal stromal cells toward a pro-inflammatory state, which in turn activates emergency myelopoiesis in functionally compromised hematopoietic stem cells (HSCs) and progenitors. This activation leads to lineage skewing and impaired regenerative capacity (123, 124).

Concurrent suppression of stem cell factor (SCF) and CXCL12 further destabilizes HSC quiescence, amplifying proliferative stress and accelerating hematopoietic aging (125). Aged HSCs overexpress Bcl11a, which enhances IL-1β secretion and establishes a pathogenic positive feedback loop. Importantly, this cascade can be reversed by IL-1 receptor antagonism or Fcer1g deletion (126), demonstrating a direct mechanistic link between tumor-induced signals and accelerated hematopoietic aging.

Collectively, tumor-driven disruption of the bone marrow niche promotes emergency myelopoiesis, HSC exhaustion, and myeloid-biased differentiation, thereby accelerating hematopoietic aging and contributing to systemic immune dysregulation.

### 3.2 Systemic senescence signaling via extracellular vesicles

Beyond the bone marrow, tumors disseminate aging signals systemically through tumor-derived extracellular vesicles (tEVs) and cytokines, propagating senescence to distant tissues and reinforcing the tumor-aging feedback loop (127). tEVs-associated PD-L1 activates CREB/STAT signaling in T cells, inducing DNA damage and lipid metabolic disturbances that culminate in T cell senescence (128). In parallel, tumor-secreted extracellular vesicles and particles (EVPs) elicit TNFα-driven chronic inflammation, directly promoting aging-related phenotypes, including hepatic metabolic dysfunction (129).

Collectively, tumor-derived vesicles act as systemic conveyors of senescence, linking localized tumor activity to widespread tissue aging and functional decline.

### 3.3 Cachexia: multi-organ failure in systemic aging

**Figure 2:**
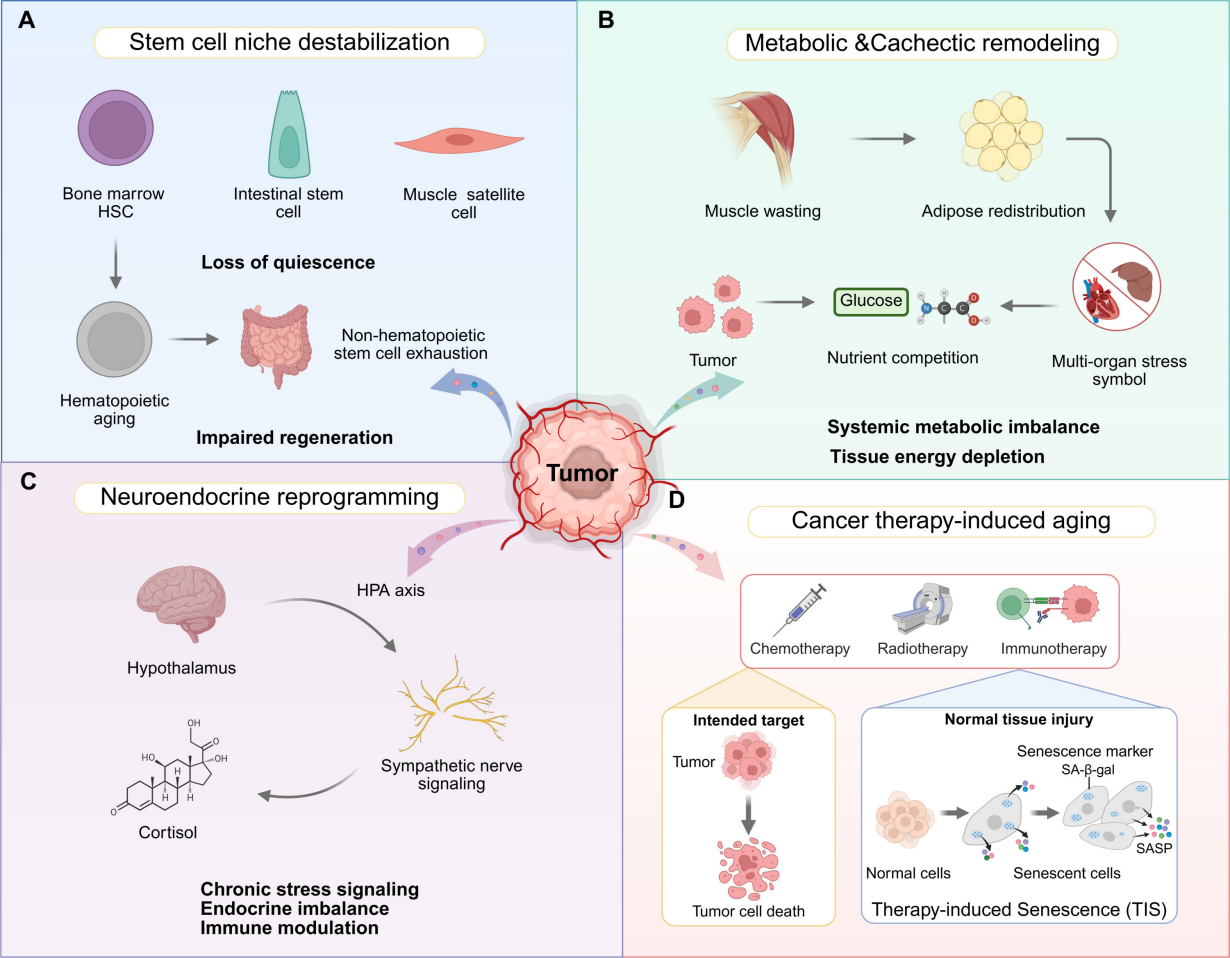
Mechanisms of tumor-accelerated aging. **(A)** Stem cell niche destabilization. Tumors disrupt the quiescence of hematopoietic and non-hematopoietic stem cells, impairing tissue regeneration and accelerating aging-related dysfunction. **(B)** Metabolic and cachectic remodeling. Tumor-driven systemic energy depletion leads to muscle wasting and adipose tissue redistribution, contributing to the development of cachexia and systemic aging. **(C)** Neuroendocrine reprogramming. Chronic activation of the hypothalamic-pituitary-adrenal (HPA) axis and sympathetic nervous system disrupts systemic homeostasis, promoting aging-related changes across multiple organ systems. **(D)** Cancer therapy-induced aging. Cytotoxic therapies (chemotherapy and radiotherapy) induce DNA damage in normal tissues, activating the p53/p21 pathway and triggering cellular senescence, which accelerates biological aging and impairs long-term tissue function. Together, these mechanisms illustrate that tumors not only arise in aging tissues but also actively drive systemic aging through multiple pathways.

Cancer-associated cachexia represents one of the most severe manifestations of tumor-driven systemic aging. Characterized by progressive loss of skeletal muscle mass, adipose tissue depletion, and metabolic dysregulation, cachexia reflects a profound catabolic state driven by tumor-host interactions (130) (**Figure 2**).

This process is mediated by both direct and indirect mechanisms. Tumor-secreted pro-inflammatory factors, including IL-6 and TNF-α, lead to upregulation of the muscle-specific E3 ubiquitin ligases MuRF1 and atrogin-1, thereby driving proteolysis and skeletal muscle atrophy (131). In parallel, tumors exert remote regulation through liver-derived factors. Dysregulation of hepatic REV-ERB alpha alters hepatokine secretion, which systemically promotes catabolic dysfunction across multiple organs. Interventional studies further demonstrate that targeting this hepatic axis can effectively reverse cachectic progression (132).

Cachexia is frequently accompanied by dysfunction of multiple organ systems, including the heart, liver, and central nervous system. Cardiac muscle atrophy, hepatic failure, and neurocognitive impairment collectively contribute to increased frailty and mortality (133, 134). From a biological perspective, cachexia represents an accelerated aging phenotype in which tumors hijack inflammatory and metabolic pathways to drive rapid organismal decline.

### 3.4 Systemic stem cell niche destabilization beyond hematopoiesis

Although aging-associated dysfunction of HSCs has been extensively studied, increasing evidence indicates that malignant transformation exerts systemic effects on non-hematopoietic stem cell compartments, including intestinal stem cells (ISCs), mesenchymal stem cells (MSCs), and skeletal muscle satellite cells (135–137). Cancer therefore acts not only as a localized pathology but also as a systemic stressor capable of disrupting stem cell niches across multiple tissues.

Tumor progression is accompanied by persistent inflammatory signaling, metabolic competition, and neuroendocrine alterations that compromise the regulatory architecture of distal stem cell microenvironments. Beyond SASP-mediated effects, tumors induce sustained niche remodeling characterized by altered cytokine gradients, increased extracellular matrix stiffness, and impaired vascular support. These changes disrupt quiescence maintenance and promote premature activation, accelerating replicative exhaustion. In cachectic mouse models, tumor burden reduces satellite cell regenerative capacity and impairs muscle repair through chronic inflammation and metabolic insufficiency (138, 139).

Metabolic stress represents an additional destabilizing axis. Rapidly proliferating tumors function as systemic nutrient sinks, altering circulating levels of glucose, amino acids, and lipids. In ISCs, nutrient deprivation combined with chronic inflammation disrupts Wnt and Notch signaling balance, reducing self-renewal capacity and compromising barrier integrity (18). In MSCs, sustained inflammatory exposure biases differentiation toward adipogenesis at the expense of osteogenesis, contributing to bone loss and frailty (140). These alterations reflect impaired functional plasticity rather than simple depletion of stem cell numbers.

Overall, tumor-induced niche destabilization lowers regenerative capacity across organs and reduces resilience to chemotherapy, infection, and physiological stress. Stem cell exhaustion thus emerges not merely as a secondary consequence of cancer, but as a parallel process that amplifies systemic vulnerability and limits the host’s ability to restrain malignant progression.

### 3.5 Tumor-driven neuroendocrine reprogramming and systemic aging acceleration

Beyond local tissue disruption, cancer significantly perturbs systemic neuroendocrine homeostasis. Chronic tumor-associated inflammation activates hypothalamic stress circuits and persistently stimulates the hypothalamic–pituitary–adrenal (HPA) axis. Elevated glucocorticoid levels suppress T-cell proliferation, impair NK-cell cytotoxicity, and promote muscle proteolysis and bone resorption. Sustained hypercortisolemia also contributes to hippocampal neuronal damage and cognitive decline observed in cancer-associated aging (141, 142).

The growth hormone (GH)-IGF-1 axis is similarly affected. Tumor-derived IGF-binding proteins and inflammatory mediators reduce systemic IGF-1 bioavailability, exacerbating sarcopenia and frailty (143). In contrast, certain malignancies aberrantly activate IGF signaling to support tumor proliferation, creating a divergence between anabolic signaling that favors tumor growth and the declining regenerative capacity of host tissues (144).

Sex hormone disruption represents a third axis of systemic dysregulation. Gonadal dysfunction, whether tumor-induced or therapy-related reduces estrogen and testosterone levels, promoting osteoporosis, endothelial dysfunction, and immune senescence (145, 146). Decreased estrogen signaling lowers antioxidant enzyme expression and increases oxidative stress, further reinforcing inflammatory pathways.

These endocrine disturbances interact closely with neural and immune circuits. Tumor-associated cytokines such as IL-1β activate vagal afferents and hypothalamic nuclei, suppress appetite, and exacerbate cachexia (147). Chronic glucocorticoid exposure contributes to thymic involution and T-cell aging (148), while sympathetic nervous system activation alters bone marrow hematopoiesis through β2-adrenergic signaling, promoting expansion of myeloid-derived suppressor cells (149).

Collectively, tumor-driven neuroendocrine reprogramming reshapes the systemic hormonal milieu and uncouples stress adaptation from tissue maintenance. This endocrine instability acts in concert with immune dysfunction and stem cell impairment, accelerating organismal aging and reducing host resilience to malignant progression.

### 3.6 Cancer therapy promotes accelerated aging

Primary anti-tumor modalities, including chemotherapy and radiotherapy, effectively control malignancy but concurrently promote organismal aging by inducing cellular senescence in normal tissues (20). These therapies accelerate biological aging primarily through DNA damage and telomere attrition (150). Chemotherapeutic agents, such as doxorubicin, inhibit topoisomerase II, causing DNA double-strand breaks that activate the p53/p21 pathway. This signaling cascade induces cell cycle arrest in normal tissues, triggering cellular senescence and subsequent tissue dysfunction. In animal models, doxorubicin treatment leads to the accumulation of senescent cells in ovarian tissue and results in irreversible fertility loss (151, 152). Similarly, γ-irradiation rapidly shortens telomeres and induces a senescent phenotype within days (153).

Beyond direct DNA damage, these therapies drive pervasive epigenetic remodeling. Tumor suppressor hypermethylation coupled with global DNA hypomethylation promotes genomic instability (154), while agents such as 5-fluorouracil alter histone acetylation, disrupt chromatin architecture, and induce persistent changes in cellular identity and function (155).

Radiation-induced senescence also remodels the tumor microenvironment. In glioblastoma, radiation upregulates tissue factor (F3) expression, creating a pro-thrombotic and pro-inflammatory niche that modulates therapeutic sensitivity. Notably, F3 can directly induce tumor cell senescence, thereby enhancing radiosensitivity and revealing a mechanism of senescence-driven tumor suppression (156). Clinical evidence further supports these findings: patients receiving radiotherapy for head and neck cancer exhibit transient epigenetic age acceleration of approximately 4.9 years, correlating with fatigue and potentially mediated by inflammatory factors such as IL-6 and IL-1ra (157).

Emerging anti-cancer modalities, including targeted therapies and certain immunotherapies, similarly accelerate aging. These agents promote senescence in hematopoietic stem cells, induce vascular dysfunction through impaired DNA repair pathways, and trigger chronic inflammatory states (158–160).

These observations indicate that both conventional and emerging anticancer therapies accelerate aging in normal tissues through multiple molecular mechanisms. Together with tumor-driven inflammatory signaling, extracellular vesicle-mediated senescence propagation, and hematopoietic remodeling, therapy-induced aging represents a central component of tumor-associated systemic aging.

Taken together, cancer should not be viewed solely as a consequence of aging tissues, but as an active accelerator of systemic aging. Through disruption of stem cell niches, neuroendocrine reprogramming, and chronic inflammatory signaling, malignancy progressively erodes regenerative capacity, hormonal stability, and immune competence.

Importantly, this interaction forms a feed-forward loop: tumor progression accelerates organismal aging, while systemic aging further diminishes constraints on malignant evolution. Cancer and aging therefore represent dynamically coupled trajectories within a shared regulatory landscape. Notably, many of the same hallmarks that predispose aged tissues to malignant transformation, including genomic instability, epigenetic dysregulation, metabolic dysfunction, chronic inflammation, immune decline, and stem cell exhaustion, are further intensified by tumors and anticancer therapies, thereby reinforcing reciprocal amplification across the tumor-aging axis. Targeting this reciprocal destabilization may offer opportunities not only to suppress tumor growth but also to preserve host resilience.

## 4 Clinical strategies: targeting the tumor-aging axis

### 4.1 Age-associated comorbidities and cancer prognosis

Targeting the tumor-aging axis is clinically relevant because aging-related processes do not merely increase cancer susceptibility, but also influence tumor progression, treatment response, and long-term clinical outcomes. In addition, tumors and anticancer therapies can further accelerate biological aging, thereby worsening host vulnerability and limiting treatment benefit. This makes the tumor-aging axis relevant not only to cancer control, but also to treatment tolerance and survivorship, particularly in older patients with a high burden of age-associated comorbidities.

One important way in which aging affects cancer prognosis is through the accumulation of age-associated comorbidities. Prognostic heterogeneity in cancer is shaped not only by chronological age, but also by frailty, functional decline, and multimorbidity (161, 162). In geriatric oncology, these aging-associated vulnerabilities are increasingly recognized as important influences on treatment tolerance and long-term outcomes (163).

Cardiometabolic comorbidities are particularly important in this context because they frequently coexist with cancer and share inflammatory, metabolic, vascular, and immune dysregulation pathways with tumor progression. These conditions are consistently associated with poorer long-term outcomes in cancer patients, with particular relevance to older adults who often carry a higher burden of multimorbidity (164, 165). For example, pre-existing cardiovascular disease has been associated with increased all-cause, cancer-specific, and cardiovascular mortality after a cancer diagnosis (166, 167). Hypertension may further compromise treatment tolerance and contribute to dose reduction, treatment delay, or incomplete delivery of anticancer therapy (168, 169). In addition, diabetes, obesity, and broader metabolic syndrome may aggravate chronic inflammatory and metabolic dysregulation, thereby contributing to poorer survival and potentially less favorable long-term outcomes (170, 171). Taken together, these findings suggest that age-associated multimorbidity is an important determinant of cancer prognosis in older patients, because it affects survival, tolerance to anticancer therapy, and long-term clinical outcomes. It should therefore be regarded as a clinically relevant determinant of outcome, rather than a secondary consideration in oncology care.

### 4.2 Biomarker-guided dynamic monitoring of biological aging

The intersection of global demographic aging with advances in understanding the tumor-aging axis has positioned therapeutic targeting of this pathway as a priority in oncology. Future translation is expected to follow a biomarker-guided, two-pronged strategy: dynamic monitoring of aging status followed by precisely targeted intervention.

Dynamic monitoring provides the diagnostic foundation for such a framework. Beyond established markers such as p16 expression and circulating SASP factors (172), next-generation biological aging clocks, including DunedinPACE and immune-specific clocks such as nTIME enable high-resolution quantification of aging pace and immune decline (173, 174).

Advanced analytical platforms further enhance precision. Spatial omics technologies, personalized physiological age modeling, and deep learning–based senescence scoring systems such as Deep-SeSMo integrate molecular and clinical variables to refine risk stratification and therapeutic timing (175–177).

**Figure 3:**
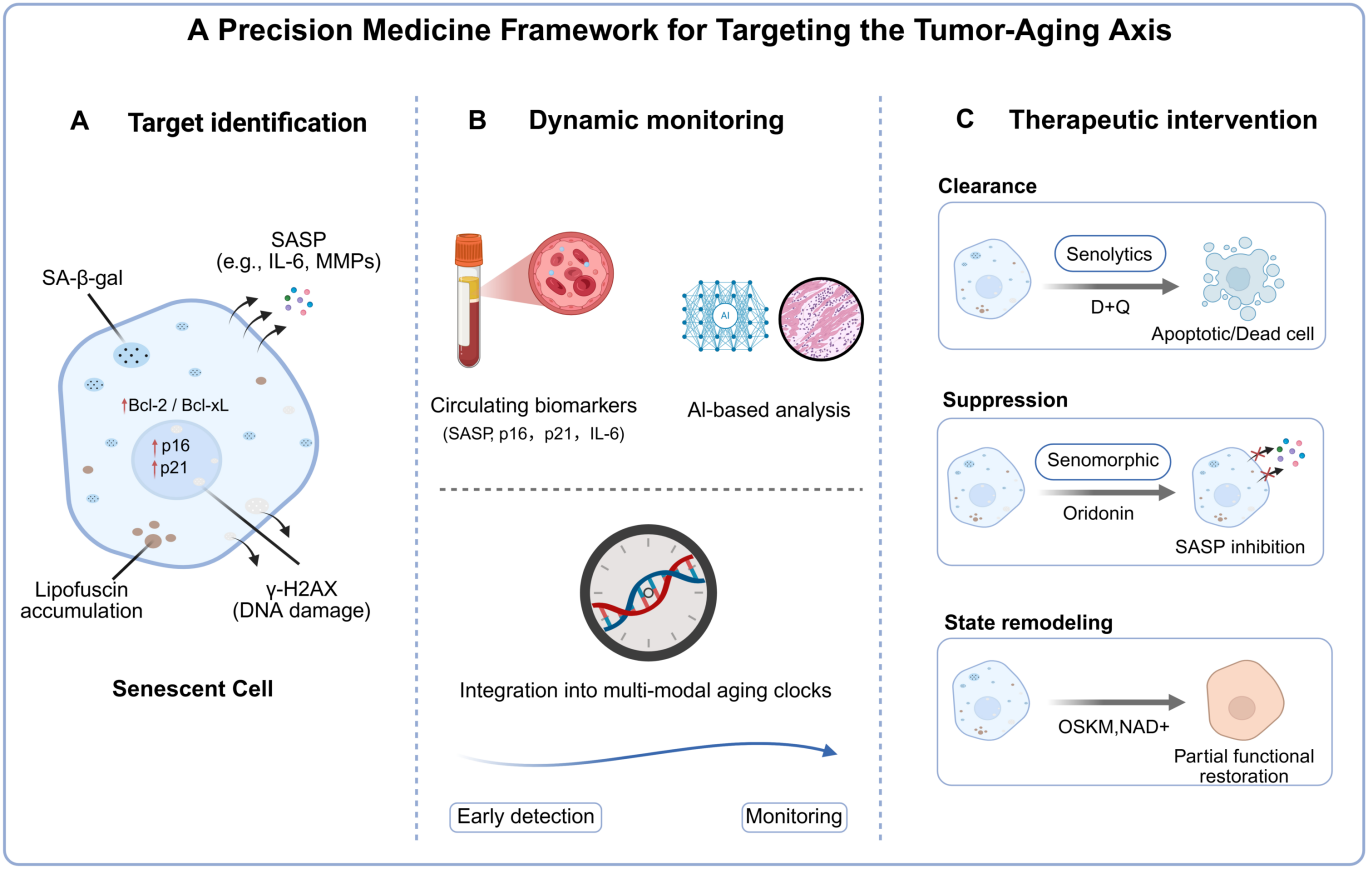
A precision medicine framework for targeting the tumor-aging axis. **(A)** Target identification. Characterization of senescent cells reveals actionable therapeutic targets, including cell-cycle arrest markers (p16, p21), pro-survival pathways (Bcl-2/Bcl-xL), lysosomal activity (SA-β-gal), lipofuscin accumulation, and DNA damage markers (γ-H2AX), which collectively distinguish senescent cells from proliferating tumor cells. **(B)** Dynamic monitoring. A multi-modal diagnostic strategy integrates circulating biomarkers derived from liquid biopsy (e.g., SASP components and senescence markers), spatial and multi-omics profiling coupled with AI-based phenotyping, and biological aging clocks to stratify patient risk, capture immune and tissue aging dynamics, and longitudinally monitor therapeutic responses. **(C)** Therapeutic intervention. A conceptual three-tiered strategy is proposed to disrupt the tumor-aging axis. Clearance: Senolytic agents (e.g., dasatinib plus quercetin) selectively eliminate senescent cells through apoptosis. Suppression: Senomorphic interventions (e.g., oridonin) attenuate the pro-tumorigenic and immunosuppressive SASP without removing senescent cells. State remodeling: Emerging metabolic and epigenetic state-remodeling approaches are conceptually incorporated to illustrate the potential reversibility of senescent phenotypes and aging-associated dysfunction. Together, these complementary strategies highlight a precision medicine framework aimed at mitigating tumor progression while alleviating systemic aging burdens.

Collectively, these approaches establish a quantitative diagnostic layer linking aging assessment with therapeutic decision-making across the cancer continuum, and are expected to further support patient selection, treatment sequencing, and on-treatment assessment for senescence-targeted interventions (**Figure 3**).

### 4.3 Senescence-targeted therapeutic strategies

Guided by this diagnostic layer, therapeutic interventions aim to modulate senescent cell burden through two complementary approaches: senolytic clearance and senomorphic suppression. A central translational challenge is that therapy-induced senescence (TIS) is biologically paradoxical. While senescence induction can initially restrain tumor growth, persistent accumulation of senescent cells after treatment may sustain SASP signaling, metabolic dysregulation, immune evasion, and therapy resistance (23, 150). Accordingly, emerging strategies increasingly aim not only to induce senescence in tumor cells, but also to eliminate or functionally reprogram persistent senescent cells in a temporally controlled manner.

Senolytics eliminate senescent cells by targeting pro-survival pathways. The dasatinib plus quercetin (D+Q) combination remains one of the best-studied first-generation regimens, and a first-in-human pilot study in idiopathic pulmonary fibrosis provided early evidence that intermittent senolytic administration is feasible in humans (178). More broadly, first-generation senolytic approaches have established proof of concept for pharmacologic clearance of senescent cells, but their use in oncology remains limited by heterogeneous pathway dependence, limited selectivity, and potential systemic toxicity (179, 180). Such safety concerns are particularly relevant for kinase-directed approaches, which may induce thromboinflammatory complications (181). To improve selectivity, more targeted platforms are being developed. For example, a micelle-encapsulated Sudan Black B conjugated analog (mGL392) has recently been reported as a selective senolytic strategy, while biologically derived approaches such as exosome-based interventions further highlight ongoing efforts to improve the precision and functional versatility of senescence-targeted therapy (182, 183).

Senomorphic strategies suppress SASP production without eliminating senescent cells. Representative approaches target inflammatory signaling nodes such as NF-κB and p38 MAPK. For example, metformin has been shown to inhibit the SASP by interfering with IKK/NF-κB activation, while p38MAPK/NF-κB-directed senomorphic strategies can protect endothelial cells from oxidative stress-mediated premature senescence (184, 185). The natural compound oridonin, for example, has been shown to reduce IL-6 and IL-8 levels by over 55% via NF-κB and p38 MAPK inhibition while preserving senescence-associated markers such as p21 (186). Compared with senolytics, senomorphic approaches may be particularly attractive when short-term senescence-associated growth arrest remains therapeutically beneficial, but persistent inflammatory signaling becomes pathogenic. However, because senescent cells are retained, the durability of benefit and the optimal duration of treatment remain important translational considerations.

### 4.4 Metabolic and epigenetic modulation

Complementary interventions target metabolic and chromatin vulnerabilities associated with aging and tumor adaptation. Tumor-associated downregulation of argininosuccinate synthase (ASS1) creates arginine auxotrophy, exploitable through arginine-degrading enzymes such as arginine deiminase or arginase (187). Epigenetic modulators, including HDAC-targeted approaches, further support chromatin regulation as a therapeutically accessible component of the tumor-aging axis, particularly in combination with cytotoxic or targeted therapies (188, 189). In parallel, the geroprotectors trametinib plus rapamycin have been shown to extend mouse healthspan and lifespan additively, illustrating the potential value of co-targeting MAPK and mTOR signaling to modify aging-associated vulnerability at the organismal level (190).

An especially promising direction is the integration of senescence-targeted approaches with conventional anticancer therapies. In a one-two-punch framework, chemotherapy, radiotherapy, or targeted therapy first drives tumor cells into senescence, after which senolytic or senomorphic interventions are applied to eliminate or restrain persistent senescent cells that would otherwise promote relapse, microenvironmental remodeling, or acquired resistance (191, 192). A recent example of this principle was provided by work showing that dual inhibition of CDK4/6 and XPO1 induces senescence together with acquired vulnerability to CRBN-based PROTAC drugs, thereby illustrating how temporally sequenced intervention may be exploited therapeutically (193).

Emerging cellular reprogramming approaches further highlight the plasticity of senescent states. Transient expression of OSKM factors has been shown to partially reverse senescence-associated phenotypes while preserving cell identity (194). In parallel, NAD^+^ precursor supplementation improves metabolic fitness and functional competence of aged cells (195).

Although these strategies remain largely preclinical, they further support the concept that senescent states are not fixed endpoints but therapeutically modifiable conditions that may be harnessed as adjuncts to tumor-directed therapy.

### 4.5 Targeting clonal hematopoietic evolution

Targeting clonal evolution in hematopoietic stem cells represents a parallel translational frontier. Epigenetic profiling and single-cell multi-omics technologies enable high-resolution mapping of aberrant HSC evolution under tumor and treatment pressures (196, 197).

In this context, monitoring clonal hematopoietic dynamics may help identify patients at risk of age-associated inflammatory remodeling, impaired hematopoietic resilience, and treatment-related functional decline (198). These insights support strategies aimed not only at eliminating malignant clones but also at restoring healthy hematopoietic function and niche integrity (199, 200).

Such approaches may ultimately complement tumor-directed senescence therapies by addressing systemic hematopoietic drivers of immune dysfunction and biological aging.

### 4.6 Future challenges and integrated closed-loop intervention

Despite these advances, significant challenges remain in drug specificity, delivery efficiency, and long-term safety. Additional barriers include incomplete biomarker standardization for patient selection and on-treatment monitoring, uncertainty regarding the optimal timing and sequencing of senotherapies relative to cancer treatment, and the risk of disrupting beneficial short-term senescence programs while attempting to suppress their chronic pathological effects. Effective translation will require integration of biomarker-based monitoring with mechanism-guided intervention to establish a closed-loop system for personalized therapy. In addition to pharmacologic interventions, healthy aging strategies such as nutritional optimization and physical activity may serve as supportive components of tumor-aging management. Although not senescence-targeted therapies in a strict sense, these approaches may help preserve metabolic resilience, reduce frailty, and improve tolerance to anticancer therapy, thereby complementing mechanism-guided interventions in older patients with cancer (201–203).

Collectively, current progress supports a dual-targeting paradigm: preventing tumor development through early modulation of senescence while concurrently mitigating aging acceleration during anticancer therapy.

In this framework, the translational value of senescence-targeted therapy lies not only in direct tumor control but also in reducing treatment-induced biological aging, improving therapeutic tolerance, and supporting long-term functional recovery. Such integration holds promise for improving treatment tolerance, quality of life, and long-term healthy aging in oncology patients.

## 5 Future directions and challenges in tumor-aging research

A major challenge in tumor-aging research is the persistent underrepresentation of aged populations in both preclinical and clinical studies. Many mechanistic insights continue to rely on young animal models, which do not fully capture the immune, metabolic, stromal, and regenerative alterations that characterize aging tissues. Similarly, older adults remain underrepresented in cancer trials, despite bearing a disproportionate burden of cancer incidence, treatment toxicity, multimorbidity, and functional decline. As a result, current evidence may not fully reflect how the tumor-aging axis operates in the populations most clinically affected (204, 205). Future progress will require wider use of age-relevant experimental models, more inclusive clinical trial design, and integration of biological aging measures into translational and clinical studies, so that therapeutic strategies can be evaluated in settings that better match real-world aging biology.

At the conceptual level, future progress will depend on more integrative frameworks capable of capturing the dynamic interaction between aging and tumor evolution. Both processes are shaped by genomic instability, epigenetic drift, immune remodeling, and metabolic adaptation, yet these factors rarely operate in isolation. Recent advances in artificial intelligence and network-based modeling have enabled the integration of epigenetic clocks, immune profiling, radiomic features, and tumor multi-omics into unified predictive systems (206, 207). Such approaches may help identify biological age acceleration, therapy tolerance, and relapse risk within a common analytical space, while also defining non-linear interactions between clonal expansion, immune decline, and metabolic instability (208). However, progress in this area will require longitudinal datasets, better harmonization of biological age estimators across tissues, and broader validation of immune-specific clocks to ensure that predictive systems become clinically informative rather than merely descriptive (209).

In parallel, experimental systems must more accurately reflect aging as a modifier of tumor biology. Conventional cancer models often overlook aging, whereas geroscience models frequently lack malignant components, leaving an important gap in the field. Organoid systems derived from aged tissues, particularly when combined with stromal and immune co-culture, provide an opportunity to investigate stem cell exhaustion, niche instability, and tumor initiation in aging context (210, 211). Spatial transcriptomics and proteomics further enable high-resolution mapping of inflammatory gradients, extracellular matrix remodeling, and cellular heterogeneity in aged tumor microenvironments (212). Emerging organ-on-chip and microfluidic platforms extend these analyses, modeling systemic interactions between tumor burden, immune aging, and organ decline (213). Further refinement of these systems, including incorporation of endocrine signaling, chronic inflammation, and long-term immune dynamics, will be essential for more faithful modeling of organismal aging.

Another unresolved question is whether certain early-onset cancers may, at least in part, reflect features of premature biological aging rather than simply earlier chronological onset. At present, this possibility should be approached cautiously. Evidence for accelerated aging is much stronger in cancer survivors, particularly childhood and adolescent/young adult survivors, who often develop early multimorbidity, functional decline, frailty, and molecular features of age acceleration (214). For *de novo* early-onset cancers, however, the available evidence remains limited and does not yet support broad generalization. Even so, findings from certain tumor types, especially early-onset colorectal cancer, suggest that biological age acceleration, inflammatory remodeling, metabolic dysregulation, and microbiota-related perturbation may contribute to tumorigenesis at younger ages (215). Overall, continued integration of biological aging clocks, multi-omics profiling, longitudinal clinical phenotyping, and age-relevant model systems will be critical for defining causal pathways within the tumor-aging axis and for translating this framework into more precise strategies for cancer prevention and treatment.
